# Supplementary material for: Misinformation effects in an online sample: results of an experimental study with a five day retention interval
Source: PeerJ. 2021 Nov 18;9:e12299. doi: 10.7717/peerj.12299 (PMC8606120; doi:10.7717/peerj.12299)
Supplement: Supplemental Information 3 [file peerj-09-12299-s003.docx]

**Data Key**

**Conditions**

**Misinfo =** Misinformation condition

**Control =** No misinformation condition

**Hits6 =** Number of hits if hits = certainty ratings >5

**Hits5 =** Number of hits if hits = certainty ratings >4

**Hits4 =** Number of hits if hits = certainty ratings >3

**Hits3 =** Number of hits if hits = certainty ratings >2

**Hits2 =** Number of hits if hits = certainty ratings >1

**Hits1 =** Number of hits if hits = certainty ratings >0

**False alarms1 =** Number of false alarms if false alarms = certainty ratings >5

**False alarms2 =** Number of false alarms if false alarms = certainty ratings >4

**False alarms3 =** Number of false alarms if false alarms = certainty ratings >3

**False alarms4 =** Number of false alarms if false alarms = certainty ratings >2

**False alarms5 =** Number of false alarms if false alarms = certainty ratings >1

**False alarms6 =** Number of false alarms if false alarms = certainty ratings >0

**H6 – H1 =** The hit rate for each cut-off criterion; calculated as HitsX/Total true items

**FA6 – FA1 =** The false alarm rate for each cut-off criterion; calculated as False alarmsX/Total misinformation items

**Adjusted hit rate =** (Hits4 + 0.5)/(Total true items +1)

**Adjusted false alarm rate =** (False alarms4 + 0.5)/(Total misinformation items + 1)

**D’ =** d-prime

**C =** bias

To calculate the area under the trapezoid (inverse of area under the curve):

**Trap 1 =** (FA6/2) x H6

**Trap 2 =** ((FA5 + FA6)/2) x (H5 – H6)

**Trap 3 =** ((FA4 + FA5)/2) x (H4 – H5)

**Trap 4 =** ((FA3 + FA4)/2) x (H3 – H4)

**Trap 5 =** ((FA2 + FA3)/2) x (H2 – H3)

**Trap 6 =** ((FA1 + FA2)/2) x (H1 – H2)

**Area =** Area of the trapezoid; Sum of trap 1 to trap 6

**AUC =** Area under the curve; inverse of trapezoid area (1-Area)

**PreVivid =** Time 1 memory vividness ratings

**PreEmo =** Time 1 memory emotionality ratings

**Attention 1 =** Attention check 1; 1 = correct, 0 = incorrect

**Attention 2 =** Attention check 2; 1 = correct, 0 = incorrect

**PostVivid =** Time 2 memory vividness ratings

**PercChangeVivid =** Perceived change in memory vividness from time 1 to time 2

**PostEmo =** Time 2 memory emotionality ratings

**PercChangeEmo =** Perceived change in memory emotionality from time 1 to time 2
